# Drug-induced upper gastrointestinal bleeding: A real-world pharmacovigilance study

**DOI:** 10.1371/journal.pone.0343209

**Published:** 2026-02-23

**Authors:** Bojing Wang, Xiaohong Wang, Shiqi Li, Yaqi Deng, Ziyan Li, Yizhou Wang, Xiaomin Shi, Wei Zhang, Fangfang Yuan, Jizhen Cai, Xiaowei Tang

**Affiliations:** 1 Department of Gastroenterology, the Affiliated Hospital of Southwest Medical University, Luzhou, China; 2 Department of Gastroenterology, Xuzhou Central Hospital, Xuzhou Clinical School of Xuzhou Medical University, Xuzhou, China; 3 Department of Intensive Care Unit, The 3rd Xiangya Hospital, Central South University, Changsha, China; 4 Department of Critical Care Medicine, Xiangya Hospital, Central South University, Changsha, China; 5 Department of Endoscopic Medicine, the Affiliated Hospital of Southwest Medical University, Luzhou, China; Lorestan University, IRAN, ISLAMIC REPUBLIC OF

## Abstract

**Background:**

Drug-induced upper gastrointestinal bleeding (UGIB) is a serious adverse event that deserves close attention. This study conducted a real-world pharmacovigilance research, aiming to enhance the understanding of drug safety and more effectively identify and prevent potential risks.

**Methods:**

This study extracted data related to UGIB reported in the Food and Drug Administration Adverse Event Reporting System (FAERS) and Japanese Adverse Drug Event Report (JADER) databases from the first quarter of 2004 to the second quarter of 2024. We selected the top 50 drugs with higher frequency and conducted safety analyses using four signal detection methods: Reporting Odds Ratio, Proportional Reporting Ratio, Empirical Bayes Geometric Mean, and Bayesian Confidence Propagation Neural Network.

**Results:**

Through data mining analysis, we found that the number of patients with UGIB reported was 62,941, including 57,414 in the FAERS and 5,527 in the JADER. It is particularly noteworthy that aspirin frequency and signal strength were among the top five in both databases. Rivaroxaban, warfarin, and pradaxa not only had the highest number of reports in the FAERS database but also showed highly in terms of their signal values. In the JADER database, clopidogrel, loxoprofen, apixaban, and bevacizumab had a higher number of reports, and it was also observed that esflurbiprofen/mentha oil and lornoxicam exhibited extremely high signal values. Meanwhile, meloxicam and prasugrel also had relatively high signal values.

**Conclusion:**

This study conducted a pharmacovigilance analysis of drug-related UGIB by integrating and analyzing multiple adverse drug reaction databases. In both the FAERS and JADER databases, we not only identified some common risk-signaling drugs but also discovered database-specific risk-associated medications. In particular, this study performed a systematic quantitative risk assessment of the selected high-reporting-frequency drugs. This analysis not only deepened our understanding of drug risk profiles but also provided important reference evidence for clinical medication safety decision-making.

## Introduction

Upper Gastrointestinal Bleeding (UGIB), defined as gastrointestinal bleeding occurring from the oral cavity to the Treitz ligament [1], represents a common emergency that poses a significant threat to patient life. Studies have shown that the incidence of UGIB in the general population ranged from 84 to 160 cases per 100,000 individuals, with a mortality rate of approximately 10% [2,3]. Although recent research has found a decline in the morbidity rate of UGIB [4], we must remain vigilant. UGIB is mainly categorized into variceal and nonvariceal gastrointestinal bleeding (GIB), with peptic ulcers identified as the predominant cause, followed by esophageal and gastric varicose veins. Other potential causes include acute erosive gastritis, upper gastrointestinal tumors, Mallory-Weiss syndrome, etc. [5–7]. It is worth noting that the use of medications is also an important factor contributing to UGIB. Therefore, timely identification and determination of the causative medications for UGIB are of paramount importance for the prevention and treatment of UGIB.

Currently, the drugs associated with UGIB primarily involve antiplatelet drugs, anticoagulants, non-steroidal anti-inflammatory drugs (NSAID) and selective serotonin reuptake inhibitors (SSRIs) [1]. Many studies are focusing on the adverse effects of UGIB associated with these specific drugs. For instance, research conducted by Mahady SE et al. indicated that aspirin may increase the risk of GIB by 60% [8]. Similarly, research by Benamouzig R et al. had demonstrated that multiple anticoagulants may also increase the risk of GIB [9]. Although these studies provide valuable insights, they are insufficient to present a comprehensive overview of all medications that may cause UGIB.

The World Health Organization (WHO) define pharmacovigilance (PV) as the discipline and related activities of identifying, evaluating, understanding, and preventing adverse reactions or any other problems associated with drug use [10]. This study aimed to conduct a systematic analysis of UGIB using pharmacovigilance methods. On one hand, it comprehensively explored and identified potential risk signals associated with drug-induced UGIB. On the other hand, it conduct an in-depth comparative analysis across multiple databases to evaluate the similarities and differences in drug risk profiles, thereby overcoming the limitations of single-source data. The goal was to develop a cross-validated drug risk list, providing more universal and specific evidence for clinical drug safety.

## Methods

### Data sources

This study was based on two publicly accessible databases: the Food and Drug Administration Adverse Event Reporting System (FAERS) and the Japanese Adverse Drug Event Report database (JADER), both of which contain spontaneously reported adverse drug events. We collected data from the first quarter (Q1) of 2004 to the second quarter (Q2) of 2024 from these two databases, focusing on adverse events for UGIB.

**Fig 1:**
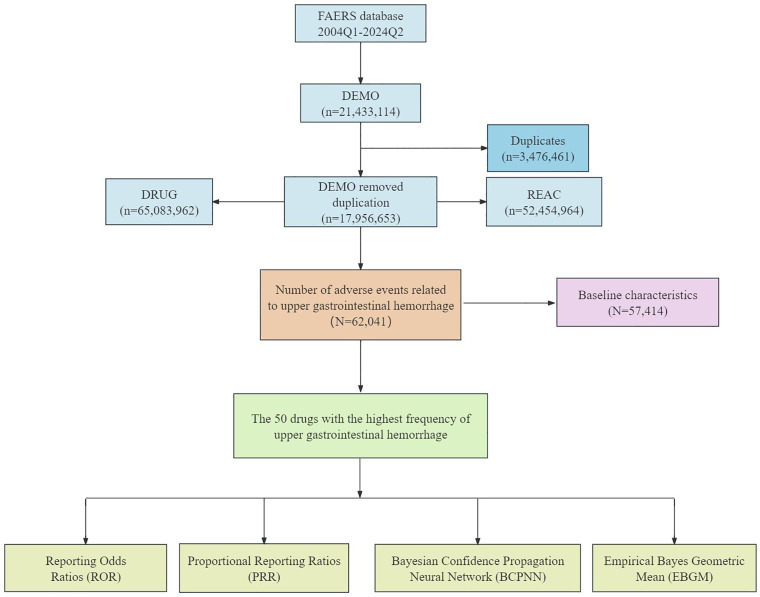
The data extraction process flowchart for drug-induced UGIB in the FAERS database.

In the FAERS database, we obtained a preliminary search of 214,331,114 related reports. After removing duplicate data, we obtained 179,566,653 unique reported cases. Further screening identified 62,041 cases related to UGIB adverse events, involving 57,414 patients. Specific data can be seen in the flowchart (Fig 1). For the JADER database, since there was no de-duplication of data, the final count involved 5,527 patients.

### Drug-induced upper gastrointestinal bleeding adverse event determination

In the FAERS and JADER databases, the adverse events involved are described using specialized medical terminology, which is standardized and categorized according to the international medical dictionary MedDRA (Medical Dictionary for Regulatory Activities). In this study, we began our analysis using the standardized medical query term “Gastrointestinal Haemorrhage” (SMQ 2000018) from the MedDRA dictionary. We then carefully filtered the terms based on anatomical location to focus on “Upper Gastrointestinal Haemorrhage”, resulting in the identification of 17 Preferred Terms (PTs). These Preferred Terms covered specific adverse events associated with UGIB, and the specific list of PTs was presented in Table 1, providing an accurate data foundation for the research.

**Table 1:** PTs for adverse events associated with UGIB.

No.	Code	PT	SOC
1	10013839	Duodenal ulcer haemorrhage	Gastrointestinal disorders
2	10013865	Duodenitis haemorrhagic	Gastrointestinal disorders
3	10017788	Gastric haemorrhage	Gastrointestinal disorders
4	10067855	Gastric occult blood positive	Investigations
5	10017826	Gastric ulcer haemorrhage	Gastrointestinal disorders
6	10017829	Gastric ulcer haemorrhage, obstructive	Gastrointestinal disorders
7	10017866	Gastritis haemorrhagic	Gastrointestinal disorders
8	10053768	Gastroduodenal haemorrhage	Gastrointestinal disorders
9	10018830	Haematemesis	Gastrointestinal disorders
10	10067786	Haemorrhagic erosive gastritis	Gastrointestinal disorders
11	10026712	Mallory-Weiss syndrome	Gastrointestinal disorders
12	10030172	Oesophageal haemorrhage	Gastrointestinal disorders
13	10030202	Oesophageal ulcer haemorrhage	Gastrointestinal disorders
14	10030219	Oesophagitis haemorrhagic	Gastrointestinal disorders
15	10034344	Peptic ulcer haemorrhage	Gastrointestinal disorders
16	10085612	Stress ulcer haemorrhage	Gastrointestinal disorders
17	10046274	Upper gastrointestinal haemorrhage	Gastrointestinal disorders

### Signal mining and statistical analysis

This study comprehensively employed four disproportionality analysis methods [11]—Reporting Odds Ratio (ROR) [12], Proportional Reporting Ratio (PRR) [13], Empirical Bayesian Geometric Mean (EBGM) [14], and Bayesian Confidence Propagation Neural Network (BCPNN) [15]—to establish a multi-dimensional cross-validation framework, integrating the strengths of each algorithm to enhance the comprehensiveness and reliability of signal detection. Based on the common framework of the 2 × 2 contingency table and by setting appropriate thresholds, this approach expanded the detection scope for potential rare adverse drug reactions while reducing the risk of false positives (S1 Table). The specific formulas and threshold criteria for each method are detailed in S2 Table. The combined multi-algorithm analysis can reveal the strength of association and the stability of signals between drugs and adverse events from different dimensions, ultimately generating a cross-validated and consistent drug risk list, thereby providing more robust evidence to support clinical medication safety.

## Results

### Clinical characteristics of patient for drug-induced upper gastrointestinal bleeding

**Fig 2:**
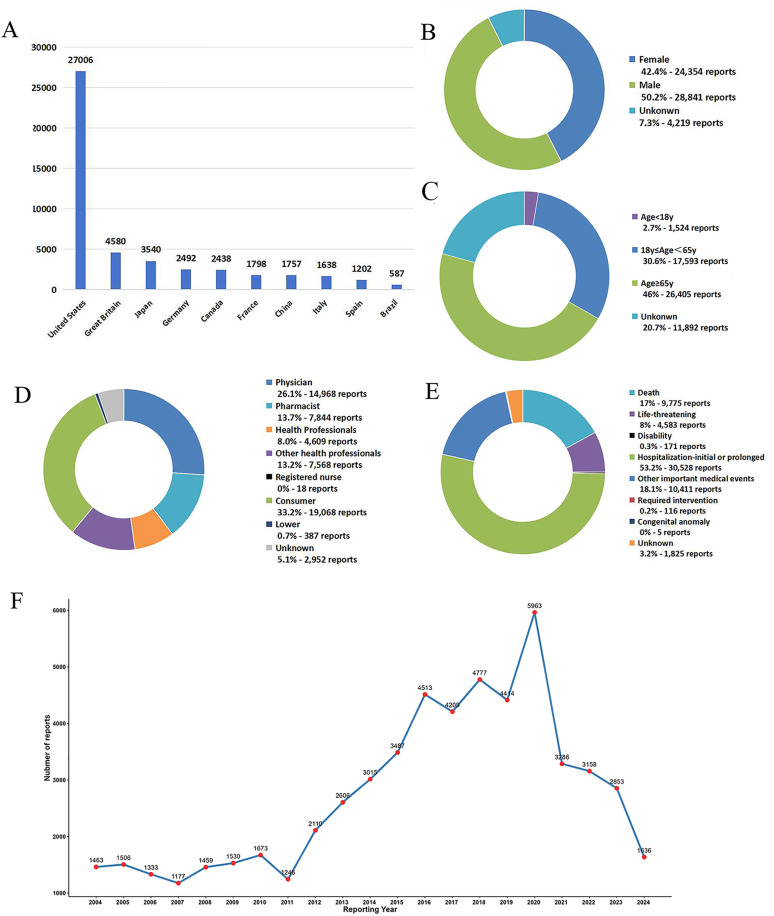
Clinical characteristics of patient for drug-induced UGIB in the FAERS database from the Q1 of 2004 to the Q2 of 2024. **(A)** Top 10 countries with the largest number of reports. **(B)** The gender distribution of patients. **(C)** The age distribution of patients. **(D)** The reporters distribution of patients. **(E)** The outcome of adverse events distribution of patients. **(F)** Line chart of the annual reports during the study period.

During the period from Q1 2004 to Q2 2024, the United States accounted for the highest number of reports (n = 27,006) of UGIB adverse events in the FAERS database (Fig 2A). Although males accounted for a relatively high proportion (50.2%), the difference in proportion between the gender was not significant (Fig 2B). The affected population was mainly concentrated in the age group of 65 years and older, accounting for 46%, followed by the age group of 18–65 years old, accounting for 30.6% (Fig 2C). Among the reporters, consumers and physicians had the highest reporting rates, at 33.2% and 26.1% respectively (Fig 2D). In terms of reported outcomes, hospitalization-initial or prolonged was the most common, with 30,528 cases, accounting for 53.2% (Fig 2E). During the study period, the highest number of reports occurred in 2020, reaching 5,963 cases (Fig 2F).

At the same time, in the FAERS database, we recorded a total of 62,041 adverse events for the 17 preferred terms involved. Among them, “Haematemesis” and “Upper Gastrointestinal Haemorrhage” had the highest proportion, with 22,731 and 15,224 cases recorded respectively, accounting for 36.64% and 24.54% of total adverse events. In comparison, “Stress Ulcer Haemorrhage” was reported relatively infrequently, constituting only 0.01% of all reports. The detailed distribution and comparison of these data can be viewed in Table 2, which revealed the frequency of reporting of different types of UGIB events.

**Table 2:** The number of UGIB reports associated with each Preferred Term.

PT	Number of reports	Percentage
Total	62,041	
Haematemesis	22,731	36.64%
Upper Gastrointestinal Haemorrhage	15,224	24.54%
Gastric Haemorrhage	10,664	17.19%
Gastric Ulcer Haemorrhage	5,172	8.34%
Duodenal Ulcer Haemorrhage	2,622	4.23%
Gastritis Haemorrhagic	1,360	2.19%
Oesophageal Haemorrhage	1,186	1.91%
Mallory-Weiss Syndrome	1,157	1.86%
Peptic Ulcer Haemorrhage	624	1.01%
Oesophageal Ulcer Haemorrhage	437	0.70%
Haemorrhagic Erosive Gastritis	309	0.50%
Oesophagitis Haemorrhagic	271	0.44%
Duodenitis Haemorrhagic	135	0.22%
Gastritis Haemorrhagic	107	0.17%
Gastric Occult Blood Positive	28	0.05%
Gastric Ulcer Haemorrhage, Obstructive	10	0.02%
Stress Ulcer Haemorrhage	4	0.01%

**Fig 3:**
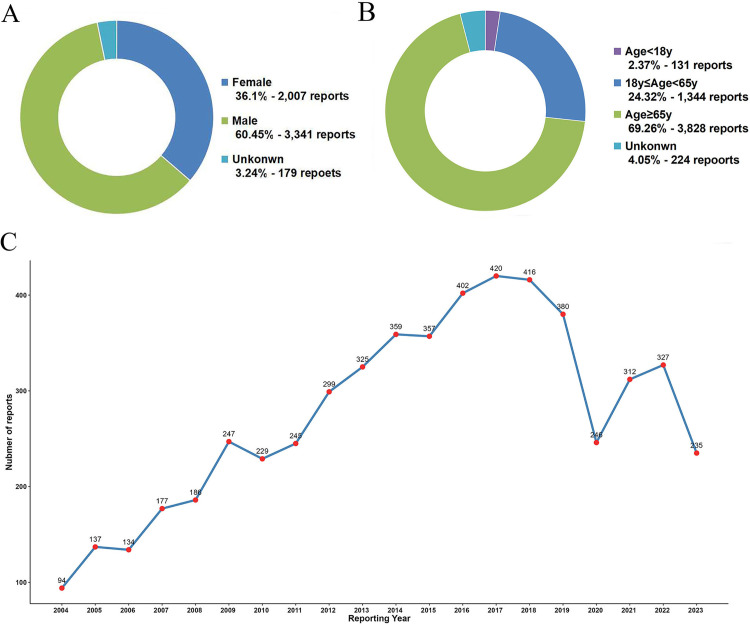
Clinical characteristics of patient for drug-induced UGIB in the JADER database from the Q1 of 2004 to the Q2 of 2024. **(A)** The gender distribution of patients. **(B)**The age distribution of patients. **(C)** Line chart of the annual reports during the study period.

In the JADER database, drug-induced UGIB adverse events were more common in males, with a total of 3,341 cases accounting for 60.45% of all reported cases (Fig 3A). The majority of these affected patients were concentrated in the age group above 65, with 3,828 cases representing 69.26% of the total reported cases. This was followed by those in the 18–65 age group at 24.32 percent (Fig 3B). From the perspective of time trend, the number of reports for UGIB generally showed an upward trend from 2004 to 2018, reaching a peak of 420 cases in 2017, while after 2019, the reported data exhibited a certain degree of fluctuation (Fig 3C).

### Drug-induced upper gastrointestinal bleeding signal analysis

**Fig 4:**
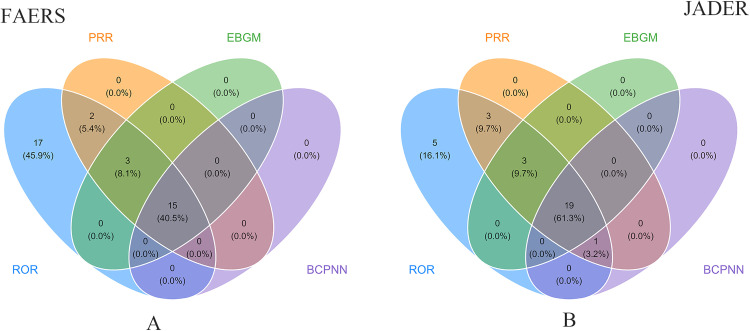
A comparative analysis of Venn diagrams for signal calculation of the top 50 drugs based on two databases. The blue part represents the number of drugs with a positive ROR signal, the orange part represents the number of drugs with a positive PRR signal, the green part shows the number of drugs with a positive EBGM signal, and the purple part refers to the number of drugs with a positive BCPNN signal. The areas with overlapping colors represent the number of drugs that are positive in different signal detection methods. **(A)** The Venn diagram of the FAERS database. **(B)** The Venn diagram of the JADER database.

In order to further explore the safety of drugs, this study carefully selected the top 50 drugs causing UGIB from the FAERS and JADER adverse drug reaction databases as research subjects. In the in-depth analysis of these identified drugs in the FAERS database, we found that 15 drugs showed positive signals across four different signal detection methods (Fig 4A), while 19 drugs showed positive signals in the JADER database (Fig 4B). Fig 4 visually showed the overlap and differences of positive signaling drugs in the two databases in the form of a venn diagram. We conducted a comprehensive and detailed signal detection and analysis of these high-risk drugs, aiming to reveal their potential safety issues.

**Fig 5:**
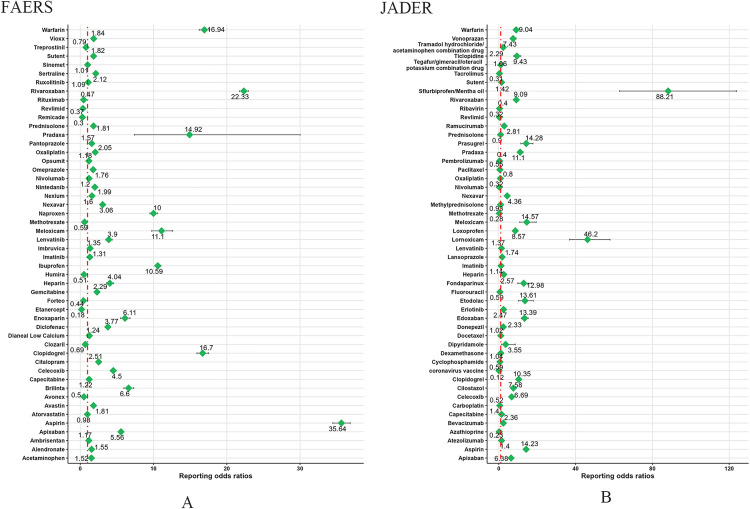
A comparative analysis of forest maps for the risk of upper gastrointestinal bleeding induced by the top 50 drugs. **(A)** The forest map of FAERS database. **(B)** The forest map of JADER database.

In the comprehensive analysis of the FAERS database, we identified the top five drugs with the highest number of reports, which were Rivaroxaban (n = 5,746), Aspirin (n = 3,680), Ibuprofen (n = 2,304), Pradaxa (n = 2,124), and Warfarin (n = 2,096). Regarding signal strength, the top five drugs assessed by ROR, PRR, EBGM, and IC values were: Aspirin (ROR 35.64, PRR 34.32, EBGM 32.24, IC 5.01), Rivaroxaban (ROR 22.33, PRR 21.83, EBGM 19.81, IC 4.31), Warfarin (ROR 16.94, PRR 16.64, EBGM 16.08, IC 4.01), Clopidogrel (ROR 16.7, PRR 16.41, EBGM 15.98, IC 4), and Pradaxa (ROR 14.92, PRR 14.67, EBGM 14.67, IC 3.88). Table 3 provided a detailed list of the signal values and reporting frequencies of these high-risk drugs for UGIB, while the forest map in Fig 5A presented the ROR values of these drugs, offering us a clear perspective to assess the safety signals of these drugs.

**Table 3:** The signal mining for the top 50 highest-frequency risk drugs associated with UGIB in the FAERS database.

Ranking	Drug	Cases	ROR(95%CI)	PRR(95%CI)	EBGM(EBGM05)	IC(IC025)
1	Rivaroxaban	5,746	22.33 (21.74 - 22.94)	21.83 (21.81-21.86)	19.81 (19.37)	4.31 (2.64)
2	Aspirin	3,680	35.64 (34.47 - 36.84)	34.32 (34.28-34.35)	32.24 (31.36)	5.01 (3.34)
3	Ibuprofen	2,304	10.59 (10.18 - 11.02)	10.48 (10.44-10.52)	10.08 (9.76)	3.33 (1.67)
4	Pradaxa	2,124	14.92 (7.41 - 30.01)	14.67 (13.99-15.30)	14.67 (8.17)	3.88 (2.2)
5	Warfarin	2,096	16.94 (16.23 - 17.68)	16.64 (16.6-16.68)	16.08 (15.52)	4.01 (2.34)
6	Clopidogrel	1,817	16.7 (15.92 - 17.53)	16.41 (16.36-16.45)	15.98 (15.35)	4 (2.33)
7	Apixaban	1,496	5.56 (5.29 - 5.85)	5.53 (5.48-5.58)	5.41 (5.19)	2.44 (0.77)
8	Humira	1,030	0.51 (0.48 - 0.55)	0.51 (0.45-0.58)	0.52 (0.49)	−0.94 (−2.61)
9	Naproxen	1,015	10 (9.51 - 10.53)	9.9 (9.85-9.95)	9.68 (9.28)	3.28 (1.61)
10	Diclofenac	1,009	3.77 (3.55 - 4)	3.76 (3.7-3.81)	3.71 (3.53)	1.89 (0.22)
11	Alendronate	576	1.55 (1.42 - 1.69)	1.55 (1.46-1.63)	1.54 (1.43)	0.62 (−1.04)
12	Celecoxib	506	4.5 (4.13 - 4.9)	4.48 (4.4-4.56)	4.45 (4.14)	2.15 (0.49)
13	Vioxx	452	1.84 (1.68 - 2.01)	1.84 (1.75-1.93)	1.83 (1.7)	0.87 (−0.79)
14	Nexium	433	1.6 (1.46 - 1.76)	1.6 (1.51-1.69)	1.6 (1.48)	0.68 (−0.99)
15	Prednisolone	405	1.81 (1.65 - 1.99)	1.81 (1.71-1.9)	1.8 (1.67)	0.85 (−0.82)
16	Avastin	398	1.81 (1.64 - 1.99)	1.8 (1.71-1.9)	1.8 (1.66)	0.85 (−0.82)
17	Sertraline	391	2.12 (1.93 - 2.33)	2.12 (2.03-2.21)	2.11 (1.95)	1.08 (−0.59)
18	Acetaminophen	382	1.52 (1.37 - 1.68)	1.52 (1.42-1.62)	1.52 (1.39)	0.6 (−1.07)
19	Brilinta	346	6.6 (5.96 - 7.31)	6.55 (6.45-6.65)	6.52 (5.99)	2.7 (1.04)
20	Methotrexate	338	0.59 (0.53 - 0.66)	0.59 (0.49-0.7)	0.6 (0.55)	−0.75 (−2.41)
21	Enoxaparin	324	6.11 (5.49 - 6.79)	6.07 (5.96-6.17)	6.04 (5.53)	2.59 (0.93)
22	Imbruvica	314	1.35 (1.21 - 1.51)	1.35 (1.24-1.46)	1.35 (1.23)	0.43 (−1.23)
23	Dianeal Low Calcium	308	1.24 (1.09 - 1.41)	1.24 (1.11-1.37)	1.24 (1.11)	0.31 (−1.36)
24	Remicade	301	0.3 (0.26 - 0.34)	0.3 (0.16-0.43)	0.3 (0.27)	−1.74 (−3.4)
25	Revlimid	294	0.37 (0.33 - 0.41)	0.37 (0.25-0.48)	0.37 (0.34)	−1.44 (−3.1)
26	Lenvatinib	293	3.9 (3.48 - 4.37)	3.89 (3.78-4)	3.88 (3.52)	1.95 (0.29)
27	Etanercept	286	0.18 (0.07-0.3)	0.18 (0.07-0.3)	0.19 (0.17)	−2.42 (−4.09)
28	Ambrisentan	276	1.17 (1.04 - 1.31)	1.17 (1.05-1.28)	1.17 (1.06)	0.22 (−1.44)
29	Sutent	276	1.82 (1.62 - 2.04)	1.81 (1.7-1.93)	1.81 (1.64)	0.86 (−0.81)
30	Avonex	272	0.5 (0.45 - 0.56)	0.5 (0.39-0.62)	0.51 (0.46)	−0.98 (−2.65)
31	Omeprazole	264	1.76 (1.56 - 1.99)	1.76 (1.64-1.88)	1.76 (1.59)	0.81 (−0.85)
32	Treprostinil	262	0.79 (0.68 - 0.93)	0.79 (0.64-0.95)	0.8 (0.7)	−0.33 (−2)
33	Nexavar	261	3.06 (2.72 - 3.44)	3.05 (2.93-3.17)	3.04 (2.76)	1.6 (−0.06)
34	Citalopram	250	2.51 (2.22 - 2.82)	2.5 (2.38-2.63)	2.49 (2.26)	1.32 (−0.35)
35	Rituximab	247	0.47 (0.42 - 0.54)	0.48 (0.35-0.6)	0.48 (0.43)	−1.07 (−2.73)
36	Heparin	243	4.04 (3.58 - 4.56)	4.02 (3.9-4.41)	4.01 (3.62)	2 (0.34)
37	Sinemet	238	1.01 (0.89 - 1.14)	1.01 (0.89-1.13)	1.01 (0.91)	0.01 (−1.65)
38	Nivolumab	235	1.2 (1.06 - 1.37)	1.2 (1.08-1.33)	1.2 (1.08)	0.27 (−1.4)
39	Oxaliplatin	235	2.05 (1.81 - 2.32)	2.04 (1.92-2.17)	2.04 (1.84)	1.03 (−0.64)
40	Imatinib	221	1.31 (1.16 - 1.49)	1.31 (1.19-1.44)	1.31 (1.18)	0.39 (−1.27)
41	Ruxolitinib	220	1.09 (0.96 - 1.23)	1.09 (0.96-1.21)	1.09 (0.98)	0.12 (−1.55)
42	Opsumit	219	1.18 (1.03 - 1.34)	1.18 (1.05-1.31)	1.18 (1.05)	0.23 (−1.43)
43	Capecitabine	215	1.22 (1.07 - 1.39)	1.22 (1.07-1.39)	1.22 (1.09)	0.29 (−1.38)
44	Atorvastatin	210	0.98 (0.85 - 1.14)	0.98 (0.84-1.13)	0.98 (0.87)	−0.02 (−1.69)
45	Meloxicam	209	11.1 (9.76 - 12.62)	10.97 (10.84-11.1)	10.93 (9.82)	3.45 (1.78)
46	Gemcitabine	197	2.29 (2 - 2.63)	2.29 (2.15-2.43)	2.29 (2.04)	1.19 (−0.47)
47	Forteo	195	0.44 (0.38 - 0.51)	0.44 (0.29-0.59)	0.44 (0.39)	−1.18 (−2.84)
48	Nintedanib	188	1.99 (1.74 - 2.28)	1.99 (1.74-2.28)	1.98 (1.77)	0.99 (−0.68)
49	Clozaril	188	0.69 (0.6 - 0.8)	0.69 (0.55-0.83)	0.69 (0.62)	−0.53 (−2.19)
50	Pantoprazole	182	1.57 (1.36 - 1.81)	1.57 (1.43-1.71)	1.57 (1.39)	0.65 (−1.02)

Table 4 comprehensively presented the results of the highest-ranking 50 drugs in the JADER database obtained through four signal analysis methods. Among these drugs, the top five with the highest frequency of occurrence were Aspirin (n = 827), Clopidogrel (n = 442), Loxoprofen (n = 428), Apixaban (n = 371), and Bevacizumab (n = 354). Furthermore, the forest plot in Fig 5B showed the ROR values of high-risk drugs, where the top five drugs with the strongest signals were, in order: Esflurbiprofen/Mentha Oil (ROR 88.21, PRR 65.91, EBGM 65.41, IC 6.03), Lornoxicam (ROR 46.2, PRR 39.35, EBGM 38.76, IC 5.28), Meloxicam (ROR 14.57, PRR 13.84, EBGM 13.73, ROR 14.57, IC 3.78), Prasugrel (ROR 14.28, PRR 13.59, EBGM 13.41, IC 3.74), Aspirin (ROR 14.23, PRR 13.61, EBGM 12.07, IC 3.59).

**Table 4:** The signal mining for the top 50 highest-frequency risk drugs associated with UGIB in the JADER database.

Ranking	Drug	Case	ROR(95%Cl)	PRR(95%Cl)	EBGM(EBGM05)	IC(IC025)
1	Aspirin	827	14.23 (13.14 - 15.41)	13.61 (12.56-14.74)	12.07 (11.14)	3.59 (1.93)
2	Clopidogrel	442	10.35 (9.31 - 11.51)	10.01 (9-11.13)	9.43 (8.48)	3.24 (1.57)
3	Loxoprofen	428	8.57 (7.74 - 9.48)	8.34 (7.54-9.22)	7.82 (7.07)	2.97 (1.3)
4	Apixaban	371	6.38 (5.68 - 7.17)	6.26 (5.57-7.03)	5.98 (5.33)	2.58 (0.91)
5	Bevacizumab	354	2.36 (2.08 - 2.67)	2.35 (2.07-2.66)	2.29 (2.02)	1.19 (−0.47)
6	Rivaroxaban	345	9.09 (8.09 - 10.21)	8.82 (7.86-9.91)	8.41 (7.49)	3.07 (1.41)
7	Warfarin	297	9.04 (7.96 - 10.27)	8.78 (7.73-9.97)	8.44 (7.43)	3.08 (1.41)
8	Edoxaban	281	13.39 (11.73 - 15.29)	12.79 (11.21-14.61)	12.32 (10.79)	3.62 (1.96)
9	Nexavar	261	4.36 (3.69 - 5.15)	4.31 (3.65-5.09)	4.23 (3.58)	2.08 (0.41)
10	Prednisolone	240	0.9 (0.77 - 1.05)	0.9 (0.77-1.05)	0.9 (0.77)	−0.15 (−1.82)
11	Pradaxa	231	11.1 (9.69 - 12.72)	10.69 (9.33-12.25)	10.32 (9.01)	3.37 (1.7)
12	Dipyridamole	214	3.55 (1.47 - 8.59)	3.52 (1.45-8.5)	3.51 (1.45)	1.81 (0.14)
13	Celecoxib	183	6.69 (5.73 - 7.82)	6.55 (5.61-7.65)	6.39 (5.47)	2.68 (1.01)
14	Dexamethasone	182	1.04 (0.84 - 1.29)	1.04 (0.84-1.92)	1.04 (0.84)	0.06 (−1.61)
15	Cilostazol	132	7.58 (6.31 - 9.1)	7.39 (6.16-8.87)	7.26 (6.05)	2.86 (1.19)
16	Capecitabine	116	1.4 (1.06 - 1.83)	1.39 (1.06-1.83)	1.39 (1.06)	0.48 (−1.19)
17	Ticlopidine	113	9.43 (7.73 - 11.5)	9.13 (7.49-11.3)	8.99 (7.37)	3.17 (1.5)
18	Vonoprazan	111	7.43 (6.08 - 9.09)	7.25 (5.93-8.87)	7.15 (5.84)	2.84 (1.17)
19	Prasugrel	110	14.28 (11.45 - 17.82)	13.59 (10.89-16.94)	13.41 (10.75)	3.74 (2.08)
20	Oxaliplatin	109	0.8 (0.62 - 1.04)	0.8 (0.62-1.04)	0.8 (0.62)	−0.32 (−1.99)
21	Azathioprine	100	0.23 (0.09 - 0.54)	0.23 (0.09-0.54)	0.23 (0.09)	−2.14 (−3.81)
22	Fluorouracil	92	0.59 (0.44 - 0.8)	0.6 (0.44-0.8)	0.6 (0.44)	−0.74 (−2.41)
23	Lornoxicam	91	46.2 (36.86 - 57.9)	39.35 (31.48-49.19)	38.76 (30.93)	5.28 (3.61)
24	Methylprednisolone	87	0.93 (0.69 - 1.25)	0.93 (0.69-1.25)	0.93 (0.69)	−0.1 (−1.77)
25	Tegafur, gimeracil, oteracil potassium	85	1.06 (0.83 - 1.36)	1.06 (0.83-1.36)	1.06 (0.83)	0.08 (−1.58)
26	Heparin	85	2.57 (2.05 - 3.23)	2.56 (2.04-3.21)	2.54 (2.02)	1.34 (−0.32)
27	Imatinib	84	1.14 (0.83 - 1.56)	1.14 (0.83-1.56)	1.13 (0.83)	0.18 (−1.48)
28	Lenvatinib	83	1.37 (1.06 - 1.77)	1.36 (1.05-1.76)	1.36 (1.05)	0.44 (−1.22)
29	Sutent	79	1.42 (1.07 - 1.9)	1.42 (1.07-1.89)	1.42 (1.06)	0.5 (−1.16)
30	Lansoprazole	68	1.74 (1.32 - 2.28)	1.73 (1.32-2.27)	1.73 (1.32)	0.79 (−0.88)
31	Ramucirumab	66	2.81 (2.19 - 3.6)	2.79 (2.17-3.58)	2.77 (2.16)	1.47 (−0.2)
32	Erlotinib	65	2.47 (1.85 - 3.3)	2.46 (1.84-3.28)	2.44 (1.83)	1.29 (−0.38)
33	Docetaxel	63	1.02 (0.78 - 1.33)	1.02 (0.78-1.33)	1.02 (0.78)	0.02 (−1.64)
34	Methotrexate	61	0.28 (0.21 - 0.37)	0.28 (0.21-0.37)	0.29 (0.22)	−1.79 (−3.46)
35	Atezolizumab	58	1.4 (1.08 - 1.8)	1.39 (1.08-1.8)	1.39 (1.08)	0.48 (−1.19)
36	Tacrolimus	55	0.31 (0.22 - 0.44)	0.31 (0.22-0.44)	0.32 (0.22)	−1.66 (−3.33)
37	Ribavirin	55	0.4 (0.29 - 0.57)	0.41 (0.29-0.57)	0.41 (0.29)	−1.29 (−2.96)
38	Revlimid	55	0.32 (0.2 - 0.51)	0.32 (0.2-0.51)	0.33 (0.2)	−1.62 (−3.29)
39	Nivolumab	55	0.32 (0.24 - 0.41)	0.32 (83.91)	0.32 (0.24-0.41)	−1.63 (−3.3)
40	Fondaparinux	54	12.98 (9.89 - 17.04)	12.41 (9.45-16.28)	12.3 (9.37)	3.62 (1.95)
41	Etodolac	51	13.61 (10.31 - 17.96)	12.97 (9.83-17.11)	12.86 (9.75)	3.69 (2.02)
42	Pembrolizumab	51	0.4 (0.31 - 0.53)	0.4 (0.31-0.53)	0.41 (0.31)	−1.29 (−2.96)
43	Carboplatin	50	0.52 (0.39 - 0.7)	0.52 (0.39-0.7)	0.53 (0.39)	−0.93 (−2.59)
44	Donepezil	50	2.33 (1.63 - 3.32)	2.32 (1.62-3.3)	2.31 (1.62)	1.21 (−0.46)
45	Paclitaxel	50	0.55 (0.41 - 0.73)	0.55 (0.41-0.73)	0.55 (0.42)	−0.86 (−2.52)
46	Coronavirus vaccine	49	0.12 (0.09 - 0.15)	0.12 (0.09-0.16)	0.13 (0.1)	−2.98 (−4.64)
47	Meloxicam	48	14.57 (10.91 - 19.45)	13.84 (10.37-18.47)	13.73 (10.28)	3.78 (2.11)
48	Cyclophosphamide	48	0.59 (0.44 - 0.8)	0.59 (0.44-0.81)	0.6 (0.44)	−0.75 (−2.41)
49	Tramadol hydrochloride/Acetaminophen combination drug	48	2.29 (1.61 - 3.26)	2.28 (1.6-3.25)	2.27 (1.59)	1.18 (−0.48)
50	Esflurbiprofen/Mentha Oil	46	88.21 (62.81 - 123.88)	65.91 (47.46-91.53)	65.41 (46.58)	6.03 (4.35)

### Outcome analysis of the high-risk drugs

**Fig 6:**
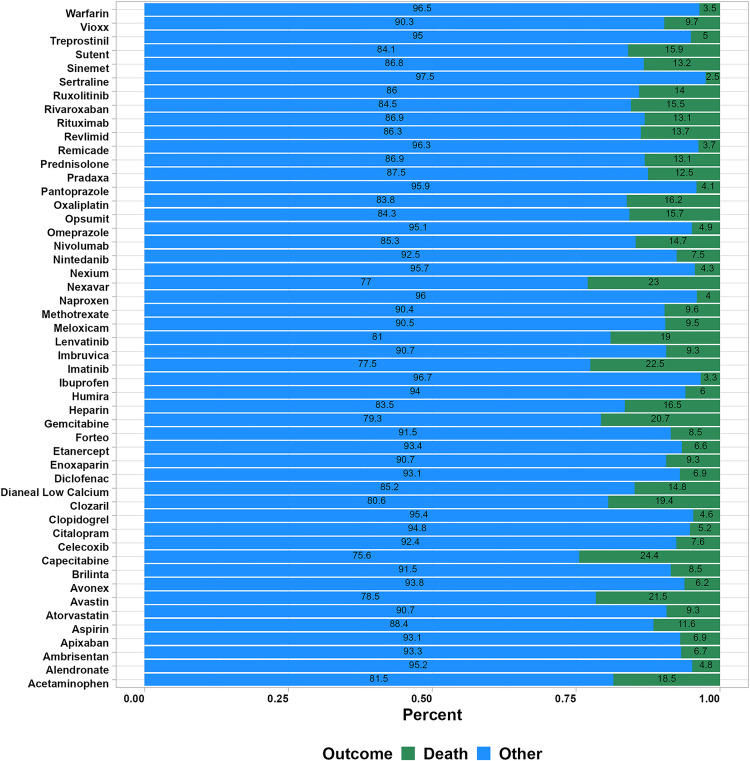
Analysis of death and non-death outcomes for the high-risk drugs inducing UGIB in the FAERS database.

By analyzing the identified risk drugs associated with UGIB in the FAERS database, we obtained a proportional distribution of death to non-death (Fig 6). The results showed that most drugs did not carry a significant risk of death. Among these drugs, capecitabine, nexavar and imatinib had relatively high mortality rates of 24.4%, 23% and 22.5%, respectively. For the JADER database, due to the small number of reports of adverse reactions to UGIB caused by target drugs, the risk of death for certain drugs may be overestimated when analyzing outcome indicators. Therefore, we did not conduct an analysis of the mortality risk following UGIB caused by drugs in the JADER database. This decision was based on the reliability of the data and the accuracy of the analysis results to ensure that our conclusions were not misleading due to small sample bias.

## Discussion

UGIB is one of the common and critical emergencies in clinical practice, frequently accompanied by a high mortality rate and a considerable healthcare burden [16]. As an important contributing factor to UGIB, the mechanisms of drug-induced bleeding are complex and hold substantial clinical significance [17]. To comprehensively explore the potential association between drugs and UGIB, this study was based on two large drug adverse reaction databases, FAERS and JADER, and combines four signal detection methods to extract data from the past two decades. A systematic analysis of drugs potentially causing UGIB was conducted, with a particular focus on identifying high-risk drugs.

This study aimed to accurately identify UGIB. Therefore, we performed a rigorous selection of PTs from an anatomical perspective. During the selection process, we excluded all PTs that could not clearly indicate the source of bleeding, such as melena, as these terms do not provide sufficient anatomical localization information and make it difficult for clinicians to determine the specific site of bleeding. Ultimately, the study included 17 PTs, among which hematemesis was observed with a higher frequency than others. This finding was consistent with previous studies regarding the clinical manifestations of UGIB, indicating that hematemesis is one of the significant indicators of UGIB [18]. Furthermore, the results revealed that the occurrence of UGIB is primarily concentrated in the stomach, suggesting that special attention should be given to gastric lesions in clinical diagnosis and treatment.

In the study of the epidemiological trends of UGIB, Abougergi MS. noted that with the discovery of Helicobacter pylori, the application of proton pump inhibitors (PPI), and the advancements of endoscopic and radiological hemostasis techniques, the incidence of UGIB had generally decreased, which was consistent with the results of most existing studies [19,20]. While these improvements had reduced ulcer-related bleeding at the population level [19], contemporary UGIB practice increasingly reflected a risk-shift toward medication-attributable bleeding, particularly in the setting of expanding exposure to antithrombotic agents and NSAIDs in aging populations [21]. This clinical transition had been highlighted in recent UGIB management updates, which emphasized drug-related impairment of gastroprotection and hemostasis as key contributors to non-variceal UGIB [22]. Consistent with prior clinical observations, our analysis also found that UGIB reports were enriched among older adults and males, a pattern that was especially evident in JADER. Therefore, given that older age and multiple medications increase bleeding risk, clinicians should assess UGIB risk and optimize prevention before starting high-risk drugs, especially antithrombotics and NSAIDs.

Across FAERS and JADER, the high-frequency drugs with consistent positive disproportionality signals clustered into four major categories—anticoagulants, antiplatelet agents, NSAIDs, and antineoplastic agents. For antithrombotic therapy, the mechanistic plausibility was direct: anticoagulants and antiplatelets increase UGIB susceptibility by impairing coagulation and platelet-mediated hemostasis, and their signals were likely amplified by high real-world exposure in cardiovascular disease care [23]. With the increasing global burden of cardiovascular diseases [24,25], the adverse event reports for these drugs have also risen. Notably, rivaroxaban, pradaxa, and warfarin, which showed strong positive signals in this study, have had their risks of inducing UGIB thoroughly confirmed in previous research [26]. NSAID-related signals were expected from known mechanisms. By inhibiting cyclooxygenase, NSAIDs reduce prostaglandin protection of the gastric mucosa. Long-term epidemiologic studies linked non-selective NSAIDs to upper GI complications, and recent meta-analyses showed that bleeding risk varies substantially between individual NSAIDs [27]. Additionally, the demand for drugs in the field of cancer treatment continued to grow, driving the development and use of antineoplastic agents [28]. In the study by Wichelmann TA et al. on bevacizumab, real cases of gastroesophageal perforation induced by the drug were identified [29]. Meanwhile, NSAIDs were often associated with UGIB due to off-label use and abuse, a problem that had long been of concern [30]. The high positive signals of these drugs in adverse reaction databases highlight the importance of continuous monitoring of their adverse reactions.

In this signal detection study, aspirin was particularly noteworthy as it showed a high frequency and signal value in the FAERS and JADER databases. Aspirin played an irreplaceable role in the medical field, exhibiting multiple beneficial effects in the treatment of cardiovascular diseases, the prevention of preeclampsia in high-risk women, and the management of small for gestational age (SGA) infants [31,32]. Recent research has increasingly indicated that aspirin can reduce the risk of colorectal cancer [33,34]. Aspirin primarily exerts its effects by irreversibly inhibiting cyclooxygenase (COX), which blocks the synthesis of prostaglandins, potentially leading to gastric mucosal damage and platelet aggregation [35]. A prospective study indicated that aspirin did not significantly enhance cardiovascular benefits in primary prevention of cardiovascular diseases but may increase the risk of UGIB [36]. Therefore, when using aspirin, we should carefully weigh its benefits against potential risks.

Esflurbiprofen/Mentha Oil was found to be a significant contributor to UGIB, primarily due to the effects of Esflurbiprofen. Esflurbiprofen is a NSAID that possesses both analgesic and anti-inflammatory properties. It is commonly prescribed for the treatment of conditions such as inflammation and osteoarthritis [37]. A study conducted by Yataba I et al. demonstrated that Esflurbiprofen, when used in the form of a plaster, exhibited a high safety profile [38]. However, this study, based on real-world data, found that Esflurbiprofen/Mentha Oil may pose a significant potential risk for UGIB when used topically, and there are currently no studies exploring its underlying mechanisms of this risk. This discovery highlights the need for further investigation by pharmaceutical researchers to better understand the possible mechanisms and fundamental principles at play.

Although this study was based on real-world data from the two major adverse drug reaction databases, FAERS and JADER, and had conducted pharmacovigilance analysis and signal detection on drugs that may cause UGIB, we must acknowledge that there were several limitations to the study. Firstly, both databases were based on spontaneous reporting systems, lacking a clear denominator, which prevent us from calculating the exact incidence rate of adverse events. Additionally, the varying levels of expertise among reporters may lead to errors such as misreporting, underreporting, or overreporting, all of which can introduce bias into the study results. Secondly, due to limitations of the databases, we cannot ascertain whether the population included in the study had underlying diseases related to the target adverse events, making it difficult to establish a precise causal relationship between the target drugs and the target adverse events. Thirdly, there were issues of racial and regional differences in the study; most of the data in FAERS came from European and American countries, while the JADER database primarily included data from the Japanese population, which may limit the generalizability of the study findings. Finally, this study did not perform statistical adjustments for potential confounding factors such as patient comorbidities or concomitant medication use, which may have influenced the observed results. Therefore, to more accurately assess the safety and efficacy of drugs associated with UGIB, future research should focus on conducting prospective multicenter studies and clinical trials, complemented by high-quality data analysis, to obtain more reliable and robust evidence.

## Conclusion

This pharmacovigilance study systematically evaluated drug-associated UGIB risks by analyzing data from the FAERS and JADER databases using multiple signal detection algorithms. The results demonstrated that anticoagulant and antiplatelet agents (including aspirin, rivaroxaban, and clopidogrel), as well as certain NSAIDs (such as esflurbiprofen/menthol oil and lornoxicam), consistently exhibited significant UGIB risk signals across both databases, with aspirin showing particularly strong consistency and representativeness. Although most UGIB events were non-fatal, a relatively higher proportion of deaths was observed in UGIB cases associated with specific anticancer agents, including capecitabine and sorafenib, indicating that their clinical risk merits careful attention. These findings provide real-world evidence to inform clinical risk assessment and monitoring, especially in patients with pre-existing bleeding risk factors, while further prospective studies and mechanistic investigations are required to confirm and elucidate these associations.

## Supporting information

S1 Table 2 × 2 contingency table structure used for disproportionality analysis. (DOCX)

S2 Table Definitions, formulas, and signal detection criteria for ROR, PRR, BCPNN, and EBGM. (DOCX)
