# Supplementary material for: Drug-induced upper gastrointestinal bleeding: A real-world pharmacovigilance study
Source: PLoS One. 2026 Feb 23;21(2):e0343209. doi: 10.1371/journal.pone.0343209 (PMC12928464; doi:10.1371/journal.pone.0343209)
Supplement: S1 Table — (DOCX) [file pone.0343209.s001.docx]

**Supplementary Table 1. 2×2 contingency table structure used for disproportionality analysis.**

|  | Upper gastrointestinal bleeding | Non-upper gastrointestinal bleeding | Total |
| --- | --- | --- | --- |
| Target drugs | a | b | a+b |
| Non-target drugs | c | d | c+d |
| Total | a+c | b+d | N=a+b+c+d |
