# Supplementary material for: Drug-induced upper gastrointestinal bleeding: A real-world pharmacovigilance study
Source: PLoS One. 2026 Feb 23;21(2):e0343209. doi: 10.1371/journal.pone.0343209 (PMC12928464; doi:10.1371/journal.pone.0343209)
Supplement: S2 Table — (DOCX) [file pone.0343209.s002.docx]

**Supplementary Table 2. Definitions, formulas, and signal detection criteria for ROR, PRR, BCPNN, and EBGM.**

| Method | Formula | Threshold |
| --- | --- | --- |
| ROR | ROR=$\frac{(a/c)}{(b/d)}=\frac{ad}{bc}$ | a≥3 and 95%CI(lower limit)>1 |
|  | SE(lnROR)=$\sqrt{(\frac{1}{a}+\frac{1}{b}+\frac{1}{c}+\frac{1}{d})}$ |  |
|  | 95%CI=℮^ln(ROR)±1.96^ |  |
| PRR | PRR= | a≥3, PRR＞2, x^2^＞4 |
|  | SE(lnPRR)= |  |
|  | 95%CI=℮^ln(PRR)±1.96^ |  |
|  | x^2^=(ad-bc)2(a+b+c+d)/[(a+b)(c+d)(a+c)(b+d)] |  |
| BCPNN | IC=log_2_=log_2_ | IC025＞0 |
|  | E(IC)=log_2_ |  |
|  | V(IC)={[+[]+[]} |  |
|  | γ=γ11 |  |
|  | IC-2SD=E(IC)-2 |  |
| EBGM | EBGM= | EBGM05＞2 |
|  | 95%CI=℮^ln（EBGM)±1.96^ |  |
